# Age-related increases in parathyroid hormone may be antecedent to both osteoporosis and dementia

**DOI:** 10.1186/1472-6823-9-21

**Published:** 2009-10-13

**Authors:** Eric R Braverman, Thomas JH Chen, Amanda LC Chen, Vanessa Arcuri, Mallory M Kerner, Anish Bajaj, Javier Carbajal, Dasha Braverman, B William Downs, Kenneth Blum

**Affiliations:** 1 Department of Neurological Surgery, Weill Cornell College of Medicine, New York, New York, USA; 2 Department of Health and Occupational Safetly, Chang Jung Christian University, Taiwan, Republic Of China; 3 Department of Engineering, Chang Jung Christian University, Taiwan, Republic Of China; 4 Department of Neurological Research, Path Research Foundation, New York, NY, USA; 5 Department of Molecular Nutrition & Nutrigenomics, LifeGen, Inc La Jolla, California, USA; 6 Department of Physiology and Pharmacology, Wake Forest University School of Medicine, Winston -Salem, NC, USA; 7 Department of Psychiatry, School of Medicine, University of Florida, Gainesville, FL, USA; 8 Path Medical Research Foundation, 304 Park Ave South, 6th Floor, NY, NY 10010, USA

## Abstract

**Background:**

Numerous studies have reported that age-induced increased parathyroid hormone plasma levels are associated with cognitive decline and dementia. Little is known about the correlation that may exist between neurological processing speed, cognition and bone density in cases of hyperparathyroidism. Thus, we decided to determine if parathyroid hormone levels correlate to processing speed and/or bone density.

**Methods:**

The recruited subjects that met the inclusion criteria (n = 92, age-matched, age 18-90 years, mean = 58.85, SD = 15.47) were evaluated for plasma parathyroid hormone levels and these levels were statistically correlated with event-related P300 potentials. Groups were compared for age, bone density and P300 latency. One-tailed tests were used to ascertain the statistical significance of the correlations. The study groups were categorized and analyzed for differences of parathyroid hormone levels: parathyroid hormone levels <30 (n = 30, mean = 22.7 ± 5.6 SD) and PTH levels >30 (n = 62, mean = 62.4 ± 28.3 SD, p ≤ 02).

**Results:**

Patients with parathyroid hormone levels <30 showed statistically significantly less P300 latency (P300 = 332.7 ± 4.8 SE) relative to those with parathyroid hormone levels >30, which demonstrated greater P300 latency (P300 = 345.7 ± 3.6 SE, p = .02). Participants with parathyroid hormone values <30 (n = 26) were found to have statistically significantly higher bone density (M = -1.25 ± .31 SE) than those with parathyroid hormone values >30 (n = 48, M = -1.85 ± .19 SE, p = .04).

**Conclusion:**

Our findings of a statistically lower bone density and prolonged P300 in patients with high parathyroid hormone levels may suggest that increased parathyroid hormone levels coupled with prolonged P300 latency may become putative biological markers of both dementia and osteoporosis and warrant intensive investigation.

## Background

While numerous studies have reported that age-induced increased parathyroid hormone (PTH) plasma levels are associated with cognitive decline [1] and dementia [2,3], little is known about the correlation that may exist between neurological processing speed and bone density in cases of hyperparathyroidism or elevated PTH. The latency of the P300 auditory evoked potential (which measures processing speed and has been shown to accurately predict memory impairment [4]) has only been studied with PTH levels in chronic renal failure, and the last work on this topic, to our knowledge, was published in 1983 [5].

PTH is anabolic in bone, but when secreted in excess it is catabolic [6]. Its levels increase with age in both genders, paralleling the incidence of osteopenia and osteopososis [7]. Recombinant human PTH 1-34 (teriparatide) is now being used as a treatment for osteoporosis, and its administration has been shown to stimulate bone formation and increase bone mineral density [8]. Interestingly, it has recently been discovered that intermittent administration of teriparatide inhibits endogenous PTH production [9], possibly via negative feedback. So far, there has not yet been any published research on teriparatide administration for hyperparathyroidism-induced osteoporosis.

Our greater understanding of PTH has led to lowering of the reference ranges. In 2003, the American Kidney Foundation recommended that levels should be kept between 35 and 70 pg/ml [10] for stage 3 chronic kidney disease, which is characterized by a glomerular filtration rate (GFR) of 30-59 mL/min/1.73 m^2^, and that an estimated 7.7% of the population suffers [11]. Currently, the acceptable reference range for parathyroid hormone (PTH) is between 10 and 60 pg/mL. It has been suggested that, where there is normal renal function and elevated serum calcium, an intact PTH concentration of >50 pg/mL strongly suggests primary hyperparathyroidism [12]. It is well established that hyperparathyroidism is responsible for changes in bone metabolism leading to a reduction in bone mineral density [13], and the National Osteoporosis Foundation lists hyperparathyroidism as a risk factor for osteoporosis [14].

The P300 wave is an event related potential that can be recorded via electroencephalograph (EEG) as a positive deflection in voltage at a latency of roughly 300 + age milliseconds [4]. The presence, magnitude, topography, and time of this signal can measure and describe processing speed. Prolonged P300 latency is an antecedent to memory loss and cognitive decline [4]. Since hyperparathyroidism has already been associated with cognitive decline [1-3], and increased P300 latency is an early measure and a better predictor of preclinical dementia than memory or mental status tests [4], we decided to determine if PTH levels correlate to processing speed and/or bone density.

## Methods

### Participants

The sample consisted of 95 patients from PATH Medical, an integrative care center and research foundation. Ages ranged between 18 and 90 years, with a mean of *M *= 58.85, *SD *= 15.47. We started with age 18 because many individuals enter adulthood with poor bone density. Furthermore, osteoporosis is considered to be a childhood disease, in that childhood and adolescence are the times when peak bone mass is established [15,16]. Missing data reduced the sample size for P300 speed of processing (P300SP) and P300 voltage (P300V) to 92. For Bone Mineral Density (BMD), missing data reduced the sample size to 74. Forty (forty-two percent) were male and fifty-six (fifty-eight percent) were female. All patients signed an approved IRB consent form based on an approval from the PATH Research Foundation IRB committee (registration # IRB00002334). Patients were made aware that their results could be used in medical research and by signing the informed consent, they volunteered to participate in this study.

In this study, analysis was conducted only on patients with complete data, (n = 92, age-matched, age 18-90 years, mean = 58.85, SD = 15.47), including measurement of intact serum PTH levels (BioReference Laboratories) between 9 AM and 3 PM, BMD, and neurological processing speed as determined by P300 latency. The P300 wave is an event-related potential that can be recorded via electroencephalograph (EEG) as a positive deflection in voltage at a latency of roughly 300 msec. We have found in previous research that the P300 wave is an accurate predictor of cognitive decline [4], and the "reference" range for P300 latency is roughly 300 + age msec. In this study, groups were categorized by patients with: PTH levels <30 (n = 30, mean = 22.7 ± 5.6 SD) and PTH levels >30 (n = 62, mean = 62.4 ± 28.3 SD, p ≤ .02). We used a Hologic Dual-Energy X-Ray Absorptiometry (DEXA) machine to measure BMD, focusing on the lumbar vertebrae and the left hip for all participants. Groups were compared for age, BMD and P300 latency.

### Statistical Analysis

One-tailed tests were used to ascertain the statistical significance of the correlations. The statistical analysis of the data was conducted in four phases. First, the PTH measure was dichotomized, with PTH values below 30 categorized as 1 (*low*), and PTH values above 30 categorized as 2 (*high*). We used 30 as the cutoff since it is close to but slightly lower than the midpoint of the current reference range (35) [12], and since 30 is clearly well below the current risk range which is not yet well defined. This step was taken to test the statistical significance of differences in P300SP, P300V and BMD between low and high levels of PTH. In addition, the BMD measure was dichotomized using a median (*Md*) split procedure (*Md *= -1.88), with BMD values equal to or lower than *Md *categorized as 1 (*low*), and values greater than *Md *categorized as 2 (*high*). This step was taken to test the statistical significance of differences in P300SP and P300V, between low and high levels of BMD.

The second phase of the analysis of the data involved the calculation of the *q*-test of normality for P300SP, P300V, BMD and age. This test was conducted to determine whether parametric or non-parametric tests of significance should be used to examine differences between the high and low levels of PTH on P300SP, P300V and BMD--and between the high and low levels of BMD on P300SP and P300V. A test of normality was also conducted for PTH.

The *q*-test is calculated by dividing the sample's standard deviation by the sample's range. The obtained *q *statistic is compared against a given range for the sample size at hand, and a *q *value falling within this range, derived on the basis of a .025 significance level [17] is interpreted as indicative that the sample distribution does not depart statistically significantly from normality. A table due to Sachs (1984) [18] provides critical ranges of values for varying sample sizes. For the present sample size of 95 for age, the *q *span, ranged between 4.17 and 6.07; for the P300SP and P300V sample size of 92, it ranged between 4.15 and 6.05; and for BMD's sample size of 74, it ranged between 4 and 5.87.

The third phase of the analysis of the data involved the calculation of the correlations among age, BMD, PTH, P300SP, and P300V.

The fourth phase of the analysis of the data involved the calculation of tests of statistical significance using the dichotomized PTH measure as the independent variable and P300SP, P300V, BMD, and age as dependent variables. Tests of statistical significance were also calculated using the dichotomized BMD measure as the independent variable and P300SP and P300V as the dependent variables. The effect sizes (*ES*) of the categorized PTH and BMD measures were calculated to ascertain the strength of association between the categorized independent and the dependent variables. Effect size is calculated as



An *ES *equal to or higher than .20 is considered empirically consequential.

## Results

The following paragraphs describe the outcomes of the tests of normality, the correlational analyses, and the analyses of differences between the levels of the dichotomized forms of PTH and BMD.

### Tests of Normality

**Figure 1:**
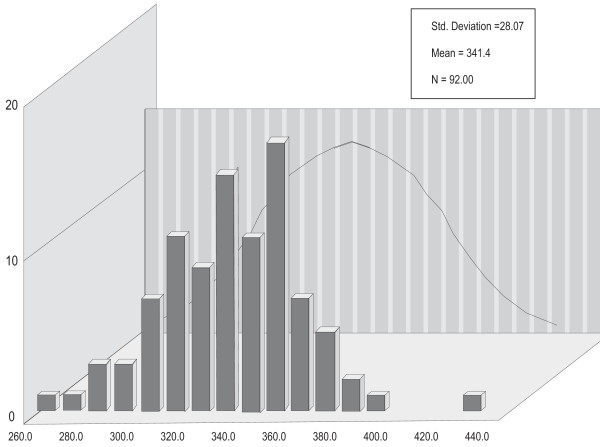
**P300 SP**.

**Figure 2:**
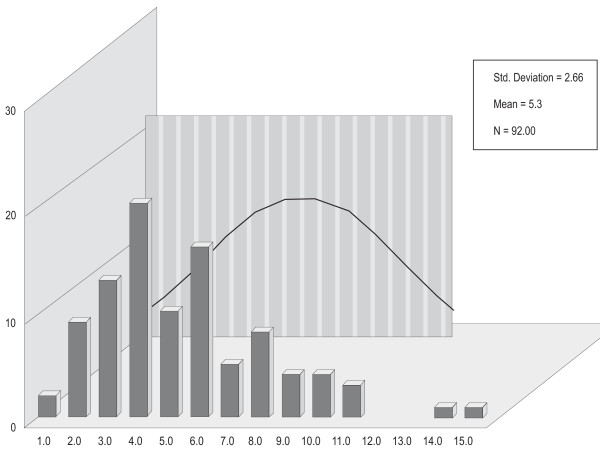
**P300 V**.

**Figure 3:**
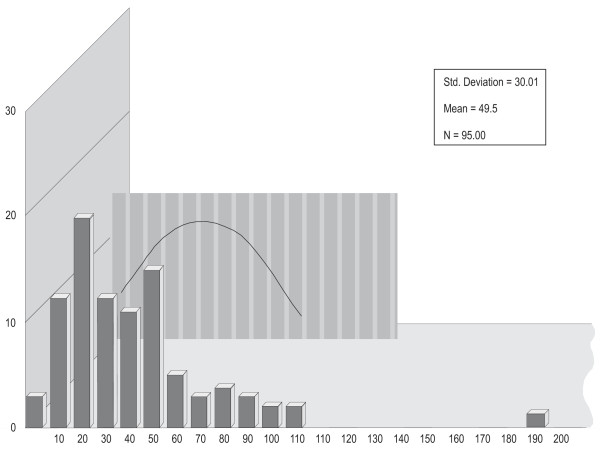
**BMD**.

**Figure 4:**
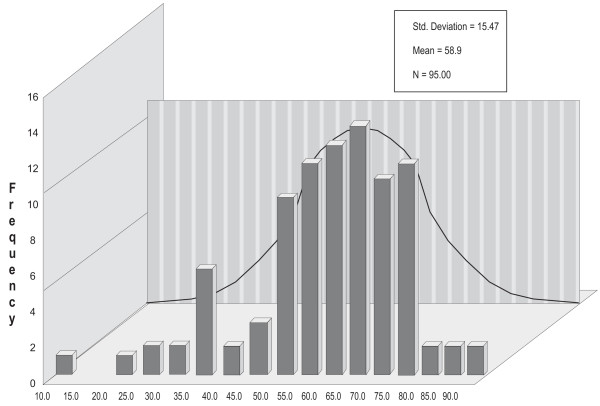
**Age**.

Figure 1 displays the distributions of P300SP, figure 2 displays the distributions of P300V, figure 3 displays the distributions of BMD, and figure 4 displays the distributions of age. For P300SP, P300V, BMD and age, the distributions tended to approach symmetry, suggesting further, more formal testing for normality by means of the *q*-test.

Table 1 displays the *q*-test outcomes. As shown in this table, the *q *value of P300SP was 6.02, that for P300V was 5.23, and that for age was 5.04--all falling within the normality range for *n *= 92 of 4.15 to 6.05. For BMD, it was 5.54, falling within the normality range for *n *= 74 of 4 to 5.87. The *q *value for PTH was 6.38, falling outside the 4.17 to 6.07 normality range for *n *= 95. These outcomes supported the assumption of normality required for the use of parametric tests in the case of P300SP, P300V, BMD, and age, and hence, the Pearson correlation coefficient was used to calculate the level of relation among the variables in continuous form, and the *t*-test was used to ascertain the statistical significance of the differences between the two categories of PTH on P300SP, P300V, BMD. Since the hypotheses were stated unidirectionally, one-tailed tests were used; alpha (a) was set at the .05 level.

**Table 1:** *q*-Tests for Normality of Distribution.

	**Variables**				
	
**Statistics**	**P300SP**	**P300V**	**BMD**	**PTH**	**Age**
***SD***	28.07	2.66	1.41	30.01	15.47
**Range**	169	13.93	7.82	191	78
***q***	6.02	5.23	5.54	6.37	5.04

The q range for normality for a sample size of 93, as was the case for P300SP, P300V, PTH, and Age, is 4.16 to 6.07 with a significance level of .025. For a sample size of 74, as was the case for Bone density, the q range is 4 to 5.87, also with a significance level of .025.

### Pearson Correlation Coefficients

Table 2 displays the Pearson correlation coefficients. In this table, BMD and PTH appear in both continuous and dichotomized form. As evidenced by the outcomes shown in this table, age proved to be statistically significantly correlated with PTH, P300SP, and P300V; BMD proved to be statistically significantly correlated with PTH in dichotomized form; BMD in dichotomized form proved to be statistically significantly correlated with P300V; PTH proved to be statistically significantly correlated with P300P; and P300SP proved to be statistically significantly correlated with P300V.

**Table 2:** Correlation Matrix.

	**1**	**2**	**3**	**4**	**5**	**6**	**7**
1. Age	1						
2. BMD	.03	1					
3. BMD^1^	.01	.62*	1				
4. PTH	.22*	-.04	-.03	1			
5. PTH^2^	.21*	-.21*	-.11	.62*	1		
6. P300SP	.40*	-.07	-.04	.18*	.22*	1	
7. P300V	-.18*	-.15	-.28*	-.06	.00	-.19*	1

^1^Dichotomized (1 <= -1.88; 1.88; 2 > -*t-Test Outcomes*-1.88) ^2^Dichotomized (1 < 30; 2 > 30)

P < 0.05

^1^Dichotomized (1 <= -* *p *< .05

The following tables depict the *t*-test outcomes. They show the means, standard deviations and standard errors of the dependent variables for each category of the independent variables, and show the *t*-statistics, *p- *values, and effects sizes.

Table 3 displays the *t*-test outcomes of P300SP by PTH in categorized form. As shown in this table, the P300SP mean of the low category of PTH (*M *= 332.66, *SD *= 6.39) proved to be statistically significantly lower than that of the high PTH category (*M *= 345.68, *SD *= 28.08), *t *= -2.12, *df *= 90, *p *= .02 (1-tail test). The effect size was *ES *= .22--an effect which, being greater than .20, proved to have practical significance according to criteria propounded by Cohen [19] and Kirk [20].

**Table 3:** *t-*Test of the Difference in P300SP Between the Categories of PTH.

	**P300SP**				***t*-Test**			
	
**PTH**	***N***	***M***	***SD***	***SE***	***T***	***Df***	***p***	ES*
1 (< 30)	30	332.66	26.39	4.81				
					-2.12	90	.02	.22
2 (> 30)	62	345.68	28.08	3.57				
Total	92	341.44	28.07	2.93				

*Effect size

These findings disclosed that level of PTH is positively associated with level of P300P, and that the strength of the association is of practical importance. The implications of these findings are discussed below.

Table 4 displays the *t-*test outcomes of P300V by PTH in categorized form. As shown in this table, the P300V mean of the low category of PTH (*M *= 5.27, *SD *= 3.15) did not differ statistically significantly from that of the high PTH category (*M *= 5.28, *SD *= 2.39), *t *= -.028, *df *= 5.31, *p *= .49 (1-tail test). The effect size was *ES *= .03, showing a null effect of PTH on P300V. These findings disclosed a lack of association between PTH and P300V levels. The implications of these findings are also discussed below.

**Table 4:** *t*-Test of the Difference in P300V Between the Categories of PTH.

	**P300V**				***t*-Test**			
**PTH**	***N***	***M***	***SD***	***SE***	***T***	***df***	***p***	ES*

1 (< 30)	30	5.27	3.15	.58			.	
					028	45.31	.49	.03
2 (> 30)	62	5.28	2.39	.30				
Total	92	5.29	2.66	.28				

*Effect size

Table 5 displays the *t-*test outcomes of BMD by PTH in categorized form. As shown in this table, the mean BMD score of participants in the low category of PTH (*M *= -1.25, *SD *= 1.57) differed statistically significantly from that of participants in the high PTH category (*M *= -1.85, *SD *= 1.28), *t *= 1.77, *df *= 72, *p *= .04 (1-tail test). The effect size was *ES *= .20, showing a substantive effect of PTH on BMD in terms of criteria propounded by [19] and [20]. These findings disclosed an empirically important association between levels of PTH and levels of BMD. The implications of these findings are also discussed below.

**Table 5:** *t*-Test of the Difference in BMD Between the Categories of PTH.

	**BMD**				***t*-Test**			
	
**PTH**	***N***	***M***	***SD***	***SE***	***T***	***Df***	***P***	ES*
1 (< 30)	26	-1.25	1.57	.31				
					1.77	72	.04	.20
2 (> 30)	48	-1.85	1.28	.19				
Total	74	-1.64	1.41	.163				

*Effect size

Table 6 displays the *t*-test outcomes for P300SP by BMD in categorized form. As shown in this table, the mean age of participants in the low category of BMD (*M *= 344.18, *SD *= 30.40) did not differ statistically significantly from that of participants in the high PTH category (*M *= 341.99, *SD *= 21.48), *t *= .35, *df *= 70, *p *= .36 (1-tail test). The effect size was *ES *= .04--an effect which, being lower than .20, proved to have insubstantial practical significance according to criteria propounded by [19] and [20]. This effect was the same as the Pearson correlation coefficient of P300SP with the original categorized BMD scores (*r *= -.04, *p *> .05). The implications of these findings are also discussed below.

**Table 6:** *t*-Test of the Difference in P300SP Between the Categories of BMD.

	**P300SP**							
	
**BMD**	***N***	***M***	***SD***	***SE***	***t***	***df***	***p***	ES*
1 (<=-1.88)	36	344.18	30.40	5.06				
					.35	70	.36	.04
2 (>-1.88)	36	341.99	21.48	3.58				
Total	92	341.44	28.07	2.93				

*Effect size

Table 7 displays the *t-*test outcomes for P300V by BMD in categorized form. As shown in this table, the mean age of participants in the low category of BMD (*M *= 5.80, *SD *= 2.83) differed statistically significantly from that of participants in the high BMD category (*M *= 4.38, *SD *= 13.66), *t *= 2.46, *df *= 70, *p *= .005 (1-tail test). The effect size was *ES *= .28--an effect which, being greater than .20, proved to have practical significance. This effect was the same as the Pearson correlation coefficient of P300SP with the original categorized BMD scores (*r *= .-.28, *p *= .008). The implications of these findings are also discussed below.

**Table 7:** *t*-Test of the Difference in P300V Between the Categories of BMD.

	**P300V**							
	
**BMD**	***N***	***M***	***SD***	***SE***	***t***	***df***	***p***	ES*
1 (<=-1.88)	36	5.80	2.83	.47				
					2.46	70	.005	.28
2 (>-1.88)	36	4.38	13.66	.33				
Total	92	5.28	2.66	.28				

Finally, table 8 displays the means, standard deviations and standard errors of the two PTH categories. As shown in this table, the two categories' means differed substantially: *category 1 (low): M *= 22.65; *category 2 (high): *62.37.

**Table 8:** Means and Standard Deviations of PTH by the Two PTH categories.

**Category**	***N***	***M***	***SD***	***SE***
1 (< 30)	30	22.65	5.62	1.03
2 (> 30)	65	62.37	28.33	3.51
Total	95	46.82	30.01	3.08

## Discussion

Our findings suggest that age dependent prolonged P300 latency, as well as age dependent increased PTH levels, may interact. This is a timely discovery since there has been a recent influx of research highlighting the connections between the brain and the bones, and a new field has been birthed called neuropsychosteology [21,22]. Many studies have confirmed neuropsychiatric disease increases with osteoporosis (OP) [23-25].

Our measurement of PTH did not take into account factors that could affect analysis: age, gender, menopausal status, vitamin D and calcium supplementation, smoking, alcohol consumption, steroid use, family history of osteoporosis, physical activity, drugs to control bone and calcium metabolism, rheumatoid arthritis, other diseases that cause secondary osteoporosis, levels of sex hormones, etc. Further studies will be needed to clarify all of these variables in relation to PTH's effects on the aging brain and aging skeleton.

Based on our findings, we suggest that control of PTH levels may be important for protecting against age-induced dementia. PTH's potential involvement with dementia may be explained in the following way: PTH has been shown to cross the blood-brain barrier [26]. PTH has been considered a candidate risk factor for senile dementia because sustained high levels of PTH in the brain may cause degeneration of specific brain regions due to Ca(2+) overloading [27,28].

Moreover, OP is a genetic disease and as such the role of 1,25-dihydroxyvitamin D_3_-receptor gene polymorphisms, known OP genetic antecedents [29] may contribute in some way to the age-linked impairments in both parathyroid and neurological processing function. It is to be noted that PTH tides are of short duration while Vitamin D or calcitriol tides are of long duration. PTH quickly mobilizes bone calcium, while calcitriol tends to more slowly increase the absorption of dietary calcium. In the case of low or no dietary calcium, calcitriol mobilizes bone calcium together with PTH. Because of this, mixed effects occur during Vitamin D deficiency, and pseudo- or secondary hyperparathyroid conditions occur. Furthermore, overnight fasting with reduced absorption of dietary calcium associated with age results in a regulatory set point inducing an increase of PTH secretion with age[30].

It is apparent that PTH levels should be kept below 60 pg/ml, and we believe based on our findings that these levels may be lowered still. Further research should involve P300 latency testing for a larger number of patients and stratification by age, and correlating PTH levels and BMD. This study provides the first potential indirect evidence that may highlight the importance of processing speed as an early electrophysiological marker of OP, which warrants further investigation.

Increases in PTH levels with age are major factors responsible for age-related increase in bone resorption, and contribute to kidney stone formation as well [31]. PTH levels need to be monitored in osteoporotic, memory-impaired people and lowering the levels may be an important part of the therapeutic process of teriparatide injections. At the PATH Medical Clinic, PTH levels were reduced by teriparatide injections by an average of 20 points, possibly due to a negative feedback mechanism. This is further supported by others [9]. Additional research has shown that teriparatide therapy may need to be supplemented by GH or GH-dependent factors in order for the anabolic response of bone [32,33].

Finally, the findings of this study showing a significant relationship between higher PTH plasma levels and prolonged P300 latency as well as a decrease in BMD suggest that hyperparathyroidism and elevated PTH due in part to age may lead to dementia and OP. The connection between elevated PTH and cognitive decline is becoming well studied. A recent study published in the Journal of Clinical Endocrinology and Metabolism presented results of various cognitive testing in postmenopausal women with hyperparathyroidism, before and after parathyroidectomy, with pre-surgical cognitive impairments improving after surgery [33]. Further studies are warranted to confirm the value of increased PTH levels coupled with increased P300 latency as putative biological markers of both dementia and OP.

## Conclusion

Patients with PTH levels <30 showed statistically significantly lesser P300 latency (P300 = 332.7 ± 4.8 SE) relative to those with high PTH levels (>30), who demonstrated greater P300 latency (P300 = 345.7 ± 3.6 SE, p = .02). In addition, participants with PTH values < 30 (n = 26) were found to have statistically significantly higher bone density (M = -1.25 ± .31 SE) than those with PTH values > 30 (n = 48, M = -1.85 ± .19 SE, p = .04)

The relevance of this research is more far reaching than might be initially suspected. A healthy skeleton is more important to overall health than for just lowering fracture risk. Healthy bones are intimately involved as an endocrine organ performing many important functions including the production of red blood cells, immune cells, platelets, various growth factors, and cytokines [35]. Bone cells and immune stem cells have a common origin and a functional relationship called the "osteo-immune relationship [35-37]" Healthy bones also exert an endocrine regulation of sugar homeostasis, fat storage, energy metabolism, cognition and more [38]. These functional relationships are the basis for the growing field of osteoimmunology [35]. Moreover, chronic immune system overexertion is known to lead to bone loss, promote muscle wasting [35] and increase fat storage [38-41].

This research supports the notion that a strong relationship exists between bone health, brain health, neurological competence, endocrine function, immune health, and genetics, building potential therapeutic bridges across the realms of neuropsychosteology, osteoimmunology, and nutrigenomics.

Our findings of a statistically lower bone density and prolonged P300 in patients with high PTH levels may suggest that PTH levels coupled with delayed P300 latency may become putative biological markers of dementia and osteoporosis, and further punctuate the importance of these relationships in the evaluation of overall mental and physical wellbeing. These conclusions support the notion that hyperparathyroidism, dysregulation of bone metabolism, osteopenia, osteoporosis, and its sub-clinical precedents have a more systemic impact on overall health, implicating the opportunity for expanding the scope of therapeutic intervention to augment treatment for other related and potentially serious disorders as well. We await further confirmation of this preliminary study.

## Competing interests

Thomas JH Chen, Amanda LC Chen, Vanessa Arcuri, Mallory Kerner, Anish Bajaj, and Javier Carbajal do not have any competing interests. We declare that we have no conflict of interest. Eric R. Braverman MD, is the director of PATH Medical, where he utilizes both the P300 and TOVA for diagnostic purposes. Kenneth Blum, PhD is the scientific director of the PATH Research Foundation and is a paid consultant, and also the chief scientific advisor for LifeGen, which granted financial support to this research. This study received funding through a grant from a private donor, Rein Narma.

## Authors' contributions

**ERB **- developed the experimental concept and design, provided editorial contributions, was clinical director of project; **TJHC**-significant editorial contributions and comments; **ALCC **- provided editorial comments and literature check for accuracy; **VA**- editorial and clinical assistance involving data collection, initiation of the first draft of the paper; **MK **- provided data collection, work on the first draft, editorial contribution, significant additions to the references and background research; **AB **-provided patient assessment and data collection; **JC **- provided patient assessment and data collection; **DB **- patient coordination, editorial comments, and obtained patient consent and regulatory involvement; **BWD **- responsible for final editorial comments; **KB **- major co -principle investigator responsible for write -up of manuscript and statistical analysis direction and paper correspondent. All authors read and approved the final manuscript.

## Pre-publication history

The pre-publication history for this paper can be accessed here:


